# Blood Pressure Management in Dialysis: A Comparative Review of Hemodialysis and Peritoneal Dialysis

**DOI:** 10.2174/0115734021408764250929235308

**Published:** 2025-10-16

**Authors:** Jonny Jonny, Nada Putri Pranidya, Adrianus Jonathan Sugiharta

**Affiliations:** 1 Department of Internal Medicine, Gatot Soebroto Central Army Hospital, Jakarta, Indonesia;; 2 Faculty of Medicine, Prima University, Medan, Indonesia;; 3 Faculty of Military Medicine, Indonesia Defense University, Bogor, Indonesia;; 4 Faculty of Medicine, Jakarta Veterans National Development University, Jakarta, Indonesia;; 5 Departmet of Cardiology and Vascular Medicine, University of Indonesia, Jakarta, Indonesia

**Keywords:** Peritoneal dialysis, hemodialysis, hypertension, chronic kidney disease, renal replacement therapy, volume management

## Abstract

Hypertension is a major challenge in patients with end-stage kidney disease (ESKD) undergoing dialysis, contributing to increased cardiovascular morbidity and mortality. Both hemodialysis (HD) and peritoneal dialysis (PD) serve as renal replacement therapies, yet their effectiveness in blood pressure (BP) control remains a subject of ongoing debate. Emerging evidence suggests that PD may offer better BP regulation than HD due to continuous ultrafiltration, superior sodium removal, and the preservation of residual kidney function. In contrast, HD is often associated with interdialytic fluid retention, leading to BP fluctuations and a higher risk of cardiovascular complications. However, BP control in PD patients is not without challenges, as ultrafiltration failure, peritoneal membrane dysfunction, and inflammation can contribute to resistant hypertension. Despite these limitations, PD may provide a more stable hemodynamic profile compared to HD, making it a promising option for patients with difficult-to-manage hypertension. Individualized dialysis selection remains crucial, as it balances the benefits of fluid control with the risks of PD-related complications. Further research, particularly randomized controlled trials, is needed to determine the long-term impact of PD and HD on BP control and cardiovascular outcomes in dialysis patients.

## 1 INTRODUCTION

Hypertension is one of the most prevalent modifiable cardiovascular risk factors in patients with end-stage kidney disease (ESKD). It affects approximately 80–90% of patients undergoing peritoneal dialysis (PD) and hemodialysis (HD) [1-3]. Poor BP control in dialysis patients is strongly associated with left ventricular hypertrophy, cardiovascular events, and increased mortality. Given that cardiovascular disease remains the leading cause of death in patients with ESKD, effective BP management is essential for improving clinical outcomes [4, 5].

Dialysis plays a crucial role in BP regulation by removing excess fluid and sodium, which are key contributors to hypertension in CKD. HD relies on intermittent ultrafiltration (UF) performed two to three times per week, often leading to BP fluctuations and interdialytic weight gain [6, 7]. In contrast, PD offers continuous UF, potentially providing more stable BP control [6, 8]. Several studies have suggested that PD patients tend to have better BP regulation due to superior volume control, sodium removal, and the preservation of residual kidney function [6-9]. However, conflicting evidence exists, and not all studies demonstrate a clear advantage of PD over HD in BP control. Concerns such as UF failure, peritoneal membrane dysfunction, and inflammation can contribute to resistant hypertension [10, 11]. Studies also have found that, in particular, patients with high peritoneal transport status, the current practice of CAPD is deemed unsuitable and may cause further comorbidities, such as fluid retention, malnutrition, and atherosclerosis [12,13].

Therefore, this review aims to critically evaluate and compare the impact of HD and PD on BP regulation, exploring the underlying mechanisms, clinical implications, and potential advantages of each modality. By analyzing current evidence, this paper seeks to provide insights that may guide clinicians in selecting the most appropriate dialysis modality for patients who are struggling with hypertension.

## 2 HYPERTENSION IN CHRONIC KIDNEY DISEASE

Hypertension is a leading cause of global disease burden and a public health threat due to its potential to cause various complications, such as stroke, coronary heart disease, and kidney failure [14,15]. Hypertension is one of the main risk factors for CKD, but CKD can also be both a common cause and consequence of inadequately treated hypertension [15,16]. Previous studies have shown a significant association between hypertension and CKD, indicating that individuals with hypertension have a 4.1 times higher risk of developing CKD compared to those without hypertension [17]. In Indonesia alone, the prevalence of hypertension in individuals aged 18 and above was recorded at 7.2% in 2007, increasing to 9.4% in 2013, and slightly decreasing to 8.36% in 2018 [18–20]. Interestingly, this trend correlates with findings from Ramadhanti et al., who identified hypertension as a strong independent risk factor for CKD (OR = 3.14; 95% CI: 1.527–6.453; *p* = 0.002), alongside age, diabetes, heart disease, and low physical activity [16].

According to the latest 2024 guidelines from the European Society of Cardiology (ESC), hypertension is defined as office systolic blood pressure (SBP) ≥140 mmHg or diastolic blood pressure (DBP) ≥90 mmHg. To confirm a hypertension diagnosis, it is recommended that BP measurements also be taken outside the office (using Home Blood Pressure Monitoring (HBPM) or Ambulatory Blood Pressure Monitoring (ABPM)) or at least verified through repeat measurements on subsequent visits. A new category called “elevated blood pressure” has been established for SBP of 120-139 mmHg or DBP of 70-89 mmHg [21]. ABPM is currently recommended for use in diagnosing and making treatment decisions for all patients with ESKD, as it offers several advantages, including superior prognostic value [4].

### 2.1 BP Measurement in CKD

**Fig. (1):**
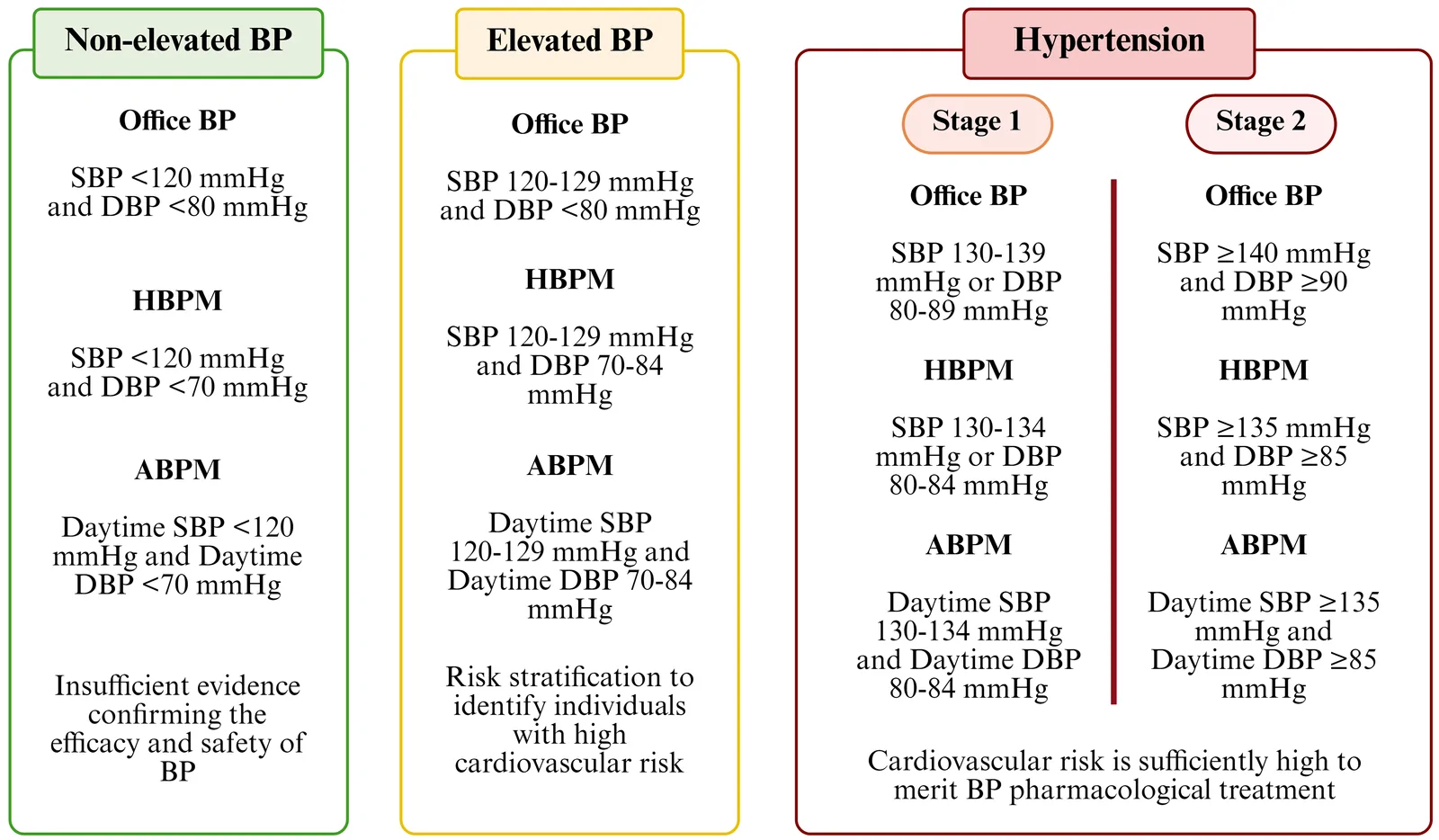
Blood Pressure Classification (modified from Williams *et al.* [28]).

Accurate BP measurement is crucial for effective hypertension management. In practice, hypertension treatment is often based on BP measurements taken in clinics or healthcare facilities [22], which can be less accurate due to the absence of repeat measurements, daily BP variations, and white coat hypertension [23]. Ambulatory Blood Pressure Monitoring (ABPM) over 24 hours provides a more precise representation of BP phenotypes and is more effective in predicting cardiovascular events in CKD patients compared to clinic-based measurements [24]. Additionally, 24-hour ABPM allows for monitoring BP fluctuations throughout the day. Home BP monitoring is a more economical alternative but should ideally be used for patients with better BP control [25]. Current hypertension guidelines, including the 2017 American College of Cardiology (ACC) guidelines (Fig. **1**), recommend out-of-office BP measurements to confirm a hypertension diagnosis and adjust antihypertensive medication doses [26]. For accuracy, only calibrated home BP devices should be used [27]. The ACC guidelines also indicate that clinic BP of 140/90 mmHg roughly corresponds to home BP of 135/85 mmHg and ABPM readings of 135/85 mmHg during the day and 120/70 mmHg at night [26].

### 2.2 Hypertension in CKD Patients Undergoing Hemodialysis

**Fig. (2):**
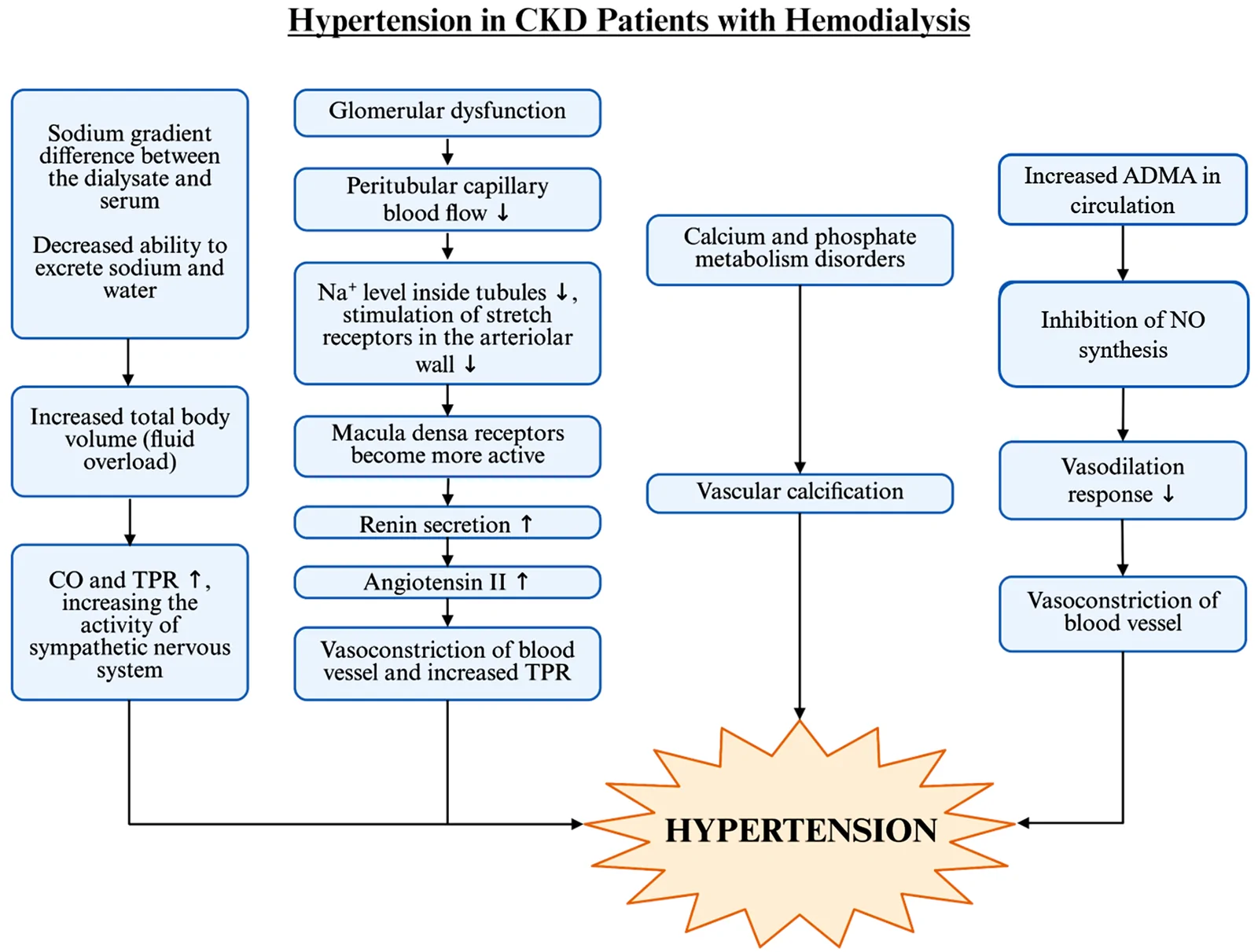
Pathophysiology of hypertension in CKD patient with HD (CO: cardiac output; TPR: total peripheral resistance; ADMA: asymmetric dimethylarginine; NO: nitric oxide).

Hypertension is a leading cause of CKD and remains a serious issue that must be addressed even after patients undergo renal replacement therapy, such as HD or PD [4]. Increased blood pressure is directly associated with lower survival rates and higher cardiovascular events. Hypertension, defined as blood pressure above 140/90 mmHg, is commonly experienced by patients undergoing regular HD, with a prevalence ranging from 50% to 60% [29]. The pathophysiology of hypertension in HD patients is complex and arises from various factors distinct from those affecting the general population (Fig. **2**). Causes of hypertension in HD include:

- **Volume Overload**: The intermittent nature of HD leads to cycles of fluid accumulation during interdialytic periods and aggressive UF during sessions, which can destabilize hemodynamics. This fluctuation activates compensatory mechanisms such as increased sympathetic nervous system activity and renin–angiotensin–aldosterone system (RAAS) stimulation, both of which raise vascular resistance and exacerbate blood pressure elevation [30-32]. Additionally, high interdialytic weight gains, often driven by excess dietary sodium and thirst induced by elevated dialysate sodium, further contribute to chronic extracellular volume expansion. While sodium is typically removed from the extracellular fluid to the dialysate during dialysis, the use of higher dialysate sodium concentrations than the patient's serum sodium can lead to a net sodium gain via diffusion. This sodium accumulation raises plasma osmolality, stimulates thirst, and subsequently promotes increased fluid intake between sessions [33, 34]. A higher sodium gradient between the dialysate and serum can increase thirst and weight gain between sessions. This fluid overload raises blood pressure through increased cardiac output and total peripheral resistance (TPR) [33, 35]. Effective management involves not only optimizing UF and accurate dry weight assessment but also carefully individualizing dialysate sodium prescriptions to avoid unnecessary sodium loading [34, 36].
- **Renin-Angiotensin-Aldosterone System (RAAS) Activation**: CKD is marked by heightened RAAS activity, driven by reduced blood flow in peritubular capillaries due to glomerular damage. This reduced blood flow leads to increased renin and aldosterone release in plasma, amplifying RAAS activity as a response to fluid shifts during dialysis. Elevated angiotensin II levels in the blood cause vasoconstriction, increasing vascular resistance and blood pressure [37-39].
- **Arterial Stiffness**: Arterial stiffness is closely linked to calcium and phosphate metabolism dysfunction, manifesting as vascular calcification, and may be influenced by inflammation or premature vascular aging. This accelerated vascular aging and arterial hardening are hallmarks of CKD progression and ESKD. It involves external remodeling of large blood vessels, marked by an increased arterial radius without proportional arterial wall hypertrophy. Structural changes, such as atherosclerosis, also thicken arterial walls, affecting blood pressure regulation [40].
- **Sympathetic Nervous System Hyperactivity**: Sympathetic nervous system hyperactivity is a well-established contributor to hypertension and cardiovascular morbidity in this particular population. Elevated muscle sympathetic nerve activity is evident even in the early stages of CKD and persists in ESRD, often independent of volume status or glomerular filtration rate [41-43]. This phenomenon is driven by a combination of enhanced renal afferent nerve signaling from ischemic or damaged nephrons, impaired baroreflex sensitivity, activation of the RAAS, and reduced nitric oxide (NO) bioavailability due to increased asymmetric dimethylarginine (ADMA) levels, an endogenous NO synthase inhibitor [40-42]. In HD patients particularly, large fluid shifts and intradialytic hypotension further amplify SNS tone [43]. Although peritoneal dialysis offers greater hemodynamic stability, SNS overactivity may still be present, especially in cases of volume overload or diminished residual kidney function [41, 43]. Notably, chronic sympathetic stimulation also contributes to vascular remodeling and arterial stiffness, further exacerbating cardiac workload and promoting left ventricular hypertrophy [40, 44].
- **Endothelial Dysfunction**: Reduced availability of nitric oxide due to decreased production and increased breakdown contributes to hypertension, further complicated by the use of erythropoietin-stimulating agents that can cause vasoconstriction [45-47].

### 2.3 Hypertension in CKD Patients Undergoing Peritoneal Dialysis

**Fig. (3):**
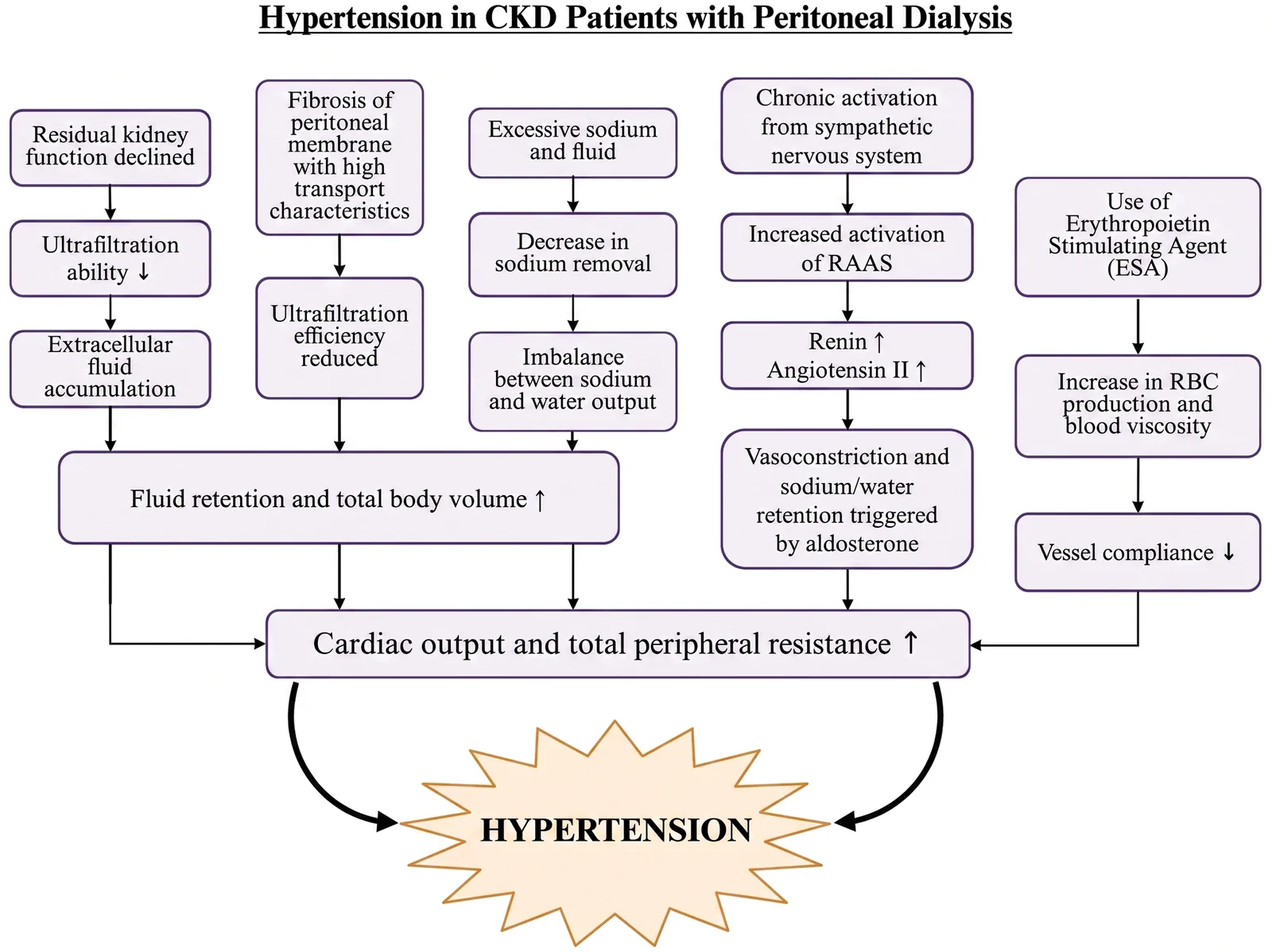
Pathophysiology of hypertension in CKD patient with peritoneal dialysis (RKF: residual kidney function; UF: ultrafiltration; ECV: extracellular fluid volume; RAAS: renin-angiotensin-aldosterone system).

Hypertension is a common health issue faced by patients undergoing PD for the treatment of ESKD. One of the primary causes of hypertension in these patients is fluid overload, often associated with the decline in residual kidney function [48]. In ESKD patients, the kidneys’ ability to excrete fluid is limited, leading to fluid accumulation in the body. Although PD is generally more effective in controlling fluid volume compared to hemodialysis, many patients still struggle with blood pressure management, particularly when their ideal dry weight is not maintained [48–50]. The pathophysiology of hypertension in PD patients (Fig. **3**) can be explained as follows:

- **Fluid Retention**: In CKD patients, fluid overload raises blood pressure not only through increased cardiac output but also by elevating systemic vascular resistance, ultimately leading to high blood pressure. It occurs due to a combination of sympathetic overactivity, maladaptive RAAS activation, endothelial dysfunction, and chronic inflammation, as explained before [40-42]. Furthermore, in patients undergoing PD, the pathophysiology of volume overload slightly differs from that of HD and is largely shaped by the continuous nature of solute and fluid exchange. It primarily results from UF failure, high peritoneal membrane transport rates, and loss of RKF, all of which impair sodium and water clearance. Unlike in HD, where volume shifts are abrupt, PD involves continuous exchange, making volume excess more insidious and difficult to detect. Loss of RKF also further exacerbates this condition and results in fluid retention and poorer blood pressure management, which is associated with a higher cardiovascular risk [47-50].
- **UF Failure**: UF failure can be defined by “the rule of fours”: failure to achieve a minimum of 400 mL of net UF during a 4-hour dwell period using 4.25% dextrose. It is a common complication in long-term PD and a significant contributor to volume overload and cardiovascular morbidity. Studies indicate that early UF failure occurs in around 4% of all PD patients, whereas late UF failure occurs in approximately 20% of patients undergoing PD for more than 2 years. It results from a spectrum of peritoneal membrane alterations that impair both free water and solute transport. A major mechanism involves a high peritoneal solute transport status, in which rapid glucose absorption shortens the duration of osmotic gradients, thereby reducing net UF. Additionally, structural changes such as peritoneal fibrosis, vasculopathy, and angiogenesis increase the vascular surface area, accelerating solute equilibration and further undermining osmotic efficiency. Another key factor is reduced osmotic conductance to glucose, which reflects impaired water transport through aquaporin-1 channels and is particularly evident in patients with low free water transport during early dwell phases. Over time, chronic exposure to hypertonic glucose solutions and peritoneal inflammation exacerbates membrane deterioration, compounding UF inefficiency. Interventions such as shorter dwell times, individualized glucose concentrations, or transitioning to automated PD may help preserve peritoneal function and maintain fluid balance in patients at risk of UF failure [51, 52].
- **Peritoneal Membrane Characteristics**: The characteristics of the peritoneal membrane play a vital role in dialysis efficacy. Variations in membrane permeability affect UF efficiency and fluid management. High-permeability membranes may require shorter exchange times but pose a risk of protein loss.
  - **Transport Type**: The type of peritoneal membrane (*e.g.*, high or low transporter) impacts UF efficiency and volume status. Patients with high peritoneal transport rates often experience suboptimal UF due to rapid glucose absorption, which leads to early dissipation of the osmotic gradient. As a result, fluid removal becomes inefficient, particularly during longer dwell times, increasing the risk of volume overload and poor blood pressure control. Tailored strategies, such as shorter dwell times or icodextrin are often required to improve fluid management in these patients [12, 13].
  - **Membrane Changes**: Long-term exposure to glucose-based solutions can cause fibrosis (scarring) of the peritoneal membrane, reducing UF efficiency and contributing to hypertension [12].
- **Sodium and Fluid Imbalance:**
  - **Sodium Intake**: High sodium consumption exacerbates fluid retention. Salt restriction is important for volume and blood pressure management.
  - **Sodium Filtration**: During the initial phase of PD exchanges with hypertonic solutions, water removal can be more rapid than sodium removal, potentially causing fluid regulation imbalances [1, 36].
- **Other Factors:**
  - **Sympathetic Nervous System Activation**: Increased sympathetic activity in PD patients can lead to vasoconstriction and higher blood pressure [40,53].
  - **Hormonal Influence**: The use of agents such as erythropoietin can increase blood viscosity and have vasoconstrictive effects, worsening hypertension [1, 53].

The volume control is better in patients with PD who undergo daily dialysis procedures compared to HD patients. However, because PD patients visit healthcare facilities less frequently than HD patients, achieving the target post-dialysis weight is easier for HD patients [54-56]. Additionally, patients with hypertension and volume overload are at high risk of cardiovascular problems, such as left ventricular hypertrophy, which can lead to cardiovascular death [57].

Overall, the mechanisms underlying hypertension in PD patients, such as volume overload from suboptimal UF, diminished residual kidney function, and high peritoneal transport rates, are consistent with the findings of this review. Several studies highlighted in our analysis reported that PD patients with impaired UF or reduced RKF were more likely to have uncontrolled blood pressure. These pathophysiological insights support the notion that volume status remains a dominant factor in PD-related hypertension. Moreover, the impact of peritoneal membrane transport type reinforces this link, as high transporters often require more intensive fluid management strategies. Therefore, by aligning the observed clinical trends with these mechanistic pathways, continuous attention and proper management approaches are necessary to improve the health and quality of life of patients with ESKD.

## 3 COMPARISON OF HYPERTENSION IN HEMODIALYSIS AND PERITONEAL DIALYSIS PATIENTS

Hypertension in dialysis patients is multifactorial, yet significant differences exist in the mechanisms, clinical manifestations, and management outcomes between those receiving HD and PD. In HD, blood pressure fluctuations are often influenced by large interdialytic weight gains and rapid UF during treatment sessions. These abrupt volume shifts can activate the sympathetic nervous system and disrupt endothelial function, leading to greater blood pressure variability and long-term cardiovascular complications. Volume overload, therefore, remains a dominant and recurring issue in HD-related hypertension, despite efforts to optimize dry weight and UF rates [30-32].

In contrast, PD allows for more continuous and gradual fluid removal, which is generally associated with more stable hemodynamics and less blood-pressure variability. Several studies suggest that PD patients may achieve better long-term blood-pressure control due to this steady-state approach [54-58]. Moreover, PD avoids the cyclic volume depletion, repletion seen in HD, contributing to reduced sympathetic activation and improved cardiovascular stability [59, 60]. However, the advantage of PD is not universal. Factors such as high peritoneal membrane transport status, diminished RKF, and suboptimal sodium removal can compromise volume control in PD patients, resulting in hypertension similar to that observed in HD [12, 13, 48, 49].

Although PD may offer advantages in terms of continuous volume control and hemodynamic stability, individual patient characteristics ultimately determine the effectiveness of blood-pressure (BP) management. Patients on dialysis are highly heterogeneous in terms of membrane transport status, dietary habits, residual renal function, and existing comorbidities such as diabetes mellitus and cardiovascular disease. These factors significantly influence volume management, UF efficiency, and therefore hypertension outcomes [12, 61, 62]. Preserving RKF has been consistently associated with better blood-pressure control in both PD and HD, owing to ongoing fluid and sodium excretion [63]. Considering this complexity, dialysis prescriptions must be tailored not only to modality but also to patient-specific risk profiles.

This review, however, is limited by the heterogeneity of included studies, differences in blood-pressure measurement techniques (office *vs*. ABPM), and the scarcity of randomized controlled trials directly comparing HD and PD for blood-pressure outcomes. Additionally, most of the cited journal articles are observational and cross-sectional studies, which may be subject to confounding and bias.

## CONCLUSION

Hypertension in chronic kidney disease patients is a complex issue that requires special management. High blood pressure can worsen kidney function, and conversely, kidney damage can trigger hypertension. PD offers several advantages in blood-pressure control compared to HD. The continuous UF process in PD allows for gradual and more stable fluid removal, helping to reduce the heart's workload and lower blood pressure (BP). Furthermore, PD is more effective in removing excess sodium, one of the main causes of hypertension. Thus, PD not only helps control blood pressure but also improves fluid and electrolyte balance in the body, and its effectiveness is highly dependent on patient adherence to therapy and proper management of fluid and sodium intake.

While PD has demonstrated potential advantages in blood pressure control, these benefits can be offset by intrinsic patient factors and peritoneal transport limitations. Future research should prioritize prospective, head-to-head studies with standardized BP metrics to more accurately delineate the comparative efficacy of HD and PD in hypertension management. Stratifying patients by RKF, transport type, and volume status may offer insights to guide more individualized and effective dialysis care.

## LIST OF ABBREVIATIONS

ESKD: End-stage Kidney Disease
HD: Hemodialysis
PD: Peritoneal Dialysis
BP: Blood Pressure
UF: Ultrafiltration
ESC: European Society of Cardiology
SBP: Systolic Blood Pressure
ABPM: Ambulatory Blood Pressure Monitoring
HBPM: Home Blood Pressure Monitoring
TPR: Total Peripheral Resistance
ADMA: Asymmetric Dimethylarginine
RAAS: Renin–angiotensin–aldosterone System
NO: Nitric Oxide
UF: Ultrafiltration
RKF: Residual Kidney Function
ECV: Extracellular Fluid Volume
CO: Cardiac Output

## References

[1] Ortega L.M., Materson B.J. (2011). Hypertension in peritoneal dialysis patients: Epidemiology, pathogenesis, and treatment.. J. Am. Soc. Hypertens..

[2] Vareta G., Georgianos P.I., Vaios V. (2022). Epidemiology of hypertension among patients on peritoneal dialysis using standardized office and ambulatory blood pressure recordings.. Am. J. Nephrol..

[3] Tsikliras N., Georgianos P.I., Vaios V. (2020). Prevalence and control of hypertension among patients on haemodialysis.. Eur. J. Clin. Invest..

[4] Sarafidis P.A., Persu A., Agarwal R. (2017). Hypertension in dialysis patients.. J. Hypertens..

[5] Sarafidis P.A., Mallamaci F., Loutradis C. (2019). Prevalence and control of hypertension by 48-h ambulatory blood pressure monitoring in haemodialysis patients: A study by the European Cardiovascular and Renal Medicine (EURECA-m) working group of the ERA-EDTA.. Nephrol. Dial. Transplant..

[6] Alexandrou M.E., Loutradis C., Schoina M. (2020). Ambulatory blood pressure profile and blood pressure variability in peritoneal dialysis compared with hemodialysis and chronic kidney disease patients.. Hypertens. Res..

[7] Flythe J.E., Chang T.I., Gallagher M.P. (2020). Blood pressure and volume management in dialysis: Conclusions from a Kidney Disease: Improving Global Outcomes (KDIGO) Controversies Conference.. Kidney Int..

[8] Kanno Y. (2021). Blood pressure management in patients receiving renal replacement therapy.. Hypertens. Res..

[9] Levin N.W., Kotanko P., Eckardt K.U. (2010). Blood pressure in chronic kidney disease stage 5D—report from a Kidney Disease: Improving Global Outcomes controversies conference.. Kidney Int..

[10] Aguirre A.R., Abensur H. (2011). Protective measures against ultrafiltration failure in peritoneal dialysis patients.. Clinics.

[11] Symonides B., Kwiatkowska-Stawiarczyk M., Lewandowski J., Małyszko J.S., Małyszko J. (2025). Resistant and apparently resistant hypertension in peritoneally dialyzed patients.. J. Clin. Med..

[12] Heaf J. (2000). Pathogenic effects of a high peritoneal transport rate.. Semin. Dial..

[13] Li P.K.T., Chow K.M. (2007). Maximizing the success of peritoneal dialysis in high transporters.. Perit. Dial. Int..

[14] (2013). Global action plan for the prevention and control of noncommunicable diseases 2013-2020.. https://iris.who.int/handle/10665/94384.

[15] Hamrahian S.M. (2017). Management of hypertension in patients with chronic kidney disease.. Curr. Hypertens. Rep..

[16] Ramadhanti R., Helda H. (2021). Association of hypertension and chronic kidney disease in population aged ≥18 years old.. Mol Cell Biomed Sci.

[17] Ingsathit A., Thakkinstian A., Chaiprasert A. (2010). Prevalence and risk factors of chronic kidney disease in the Thai adult population: Thai SEEK study.. Nephrol. Dial. Transplant..

[18] (2007). Report on Result of National Basic Health Research (RISKESDAS) 2007.. https://repository.badankebijakan.kemkes.go.id/id/eprint/4386/1/Report%20on%20Result%20of%20National%20Basic%20Health%20Research%202007.pdf.

[19] (2013). Report on Result of National Basic Health Research (RISKESDAS) 2013.. https://repository.badankebijakan.kemkes.go.id/id/eprint/4519/1/Basic_Health_Research_Riskesdas.pdf.

[20] (2018). Basic Health Research (Riskesdas) 2018.. https://layanandata.kemkes.go.id/katalog-data/riskesdas/ketersediaan-data/riskesdas-2018.

[21] McEvoy J.W., McCarthy C.P., Bruno R.M. (2024). 2024 ESC Guidelines for the management of elevated blood pressure and hypertension.. Eur. Heart J..

[22] Davis T.K., Davis A.J. (2013). Ambulatory blood pressure monitoring should be used in the primary care setting to diagnose hypertension.. Am. J. Hypertens..

[23] Sebo P., Pechère-Bertschi A., Herrmann F.R., Haller D.M., Bovier P. (2014). Blood pressure measurements are unreliable to diagnose hypertension in primary care.. J. Hypertens..

[24] Minutolo R., Gabbai F.B., Agarwal R. (2014). Assessment of achieved clinic and ambulatory blood pressure recordings and outcomes during treatment in hypertensive patients with CKD: A multicenter prospective cohort study.. Am. J. Kidney Dis..

[25] Cohen D.L., Huan Y., Townsend R.R. (2014). Home blood pressure monitoring in CKD.. Am. J. Kidney Dis..

[26] Whelton P.K., Carey R.M., Aronow W.S. (2018). 2017 ACC/AHA/AAPA/ABC/ACPM/AGS/APhA/ASH/ASPC/NMA/PCNA guideline for the prevention, detection, evaluation, and management of high blood pressure in adults: Executive summary: A report of the American college of Cardiology/American heart association task force on clinical practice guidelines.. Hypertension.

[27] Ringrose J.S., Polley G., McLean D., Thompson A., Morales F., Padwal R. (2017). An assessment of the accuracy of home blood pressure monitors when used in device owners.. Am. J. Hypertens..

[28] Williams B., Mancia G., Spiering W. (2018). 2018 ESC/ESH Guidelines for the management of arterial hypertension.. Eur. Heart J..

[29] Bucharles S.G.E., Wallbach K.K.S., Moraes T.P., Pecoits-Filho R. (2019). Hypertension in patients on dialysis: Diagnosis, mechanisms, and management.. J. Bras. Nefrol..

[30] Nongnuch A., Campbell N., Stern E., El-Kateb S., Fuentes L., Davenport A. (2015). Increased postdialysis systolic blood pressure is associated with extracellular overhydration in hemodialysis outpatients.. Kidney Int..

[31] Fishbane S., Natke E., Maesaka J.K. (1996). Role of volume overload in dialysis-refractory hypertension.. Am. J. Kidney Dis..

[32] Lins R.L., Elseviers M., Rogiers P. (1997). Importance of volume factors in dialysis related hypertension.. Clin. Nephrol..

[33] Flythe J.E., Bansal N. (2019). The relationship of volume overload and its control to hypertension in hemodialysis patients.. Semin. Dial..

[34] Loutradis C., Sarafidis P.A., Ferro C.J., Zoccali C. (2021). Volume overload in hemodialysis: Diagnosis, cardiovascular consequences, and management.. Nephrol. Dial. Transplant..

[35] Bansal N., Artinian N.T., Bakris G. (2023). Hypertension in patients treated with in-center maintenance hemodialysis: Current evidence and future opportunities: A scientific statement from the American Heart Association.. Hypertension.

[36] Van Buren P.N. (2016). Evaluation and treatment of hypertension in end-stage renal disease patients on hemodialysis.. Curr. Cardiol. Rep..

[37] Kornerup H.J., Schmitz O., Danielsen H., Pedersen E.B., Giese J., Jahn H., Massry S.G., Ritz E., Weidmann P. (1985). Significance of the renin-angiotensin system for blood pressure regulation in end-stage renal disease.. Contributions to Nephrology..

[38] Henrich W.L., Katz F.H., Molinoff P.B., Schrier R.W. (1977). Competitive effects of hypokalemia and volume depletion on plasma renin activity, aldosterone and catecholamine concentrations in hemodialysis patients.. Kidney Int..

[39] Bazzato G., Coli U., Landini S., Lucatello S., Fracasso A., Morachiello P., Jahn H., Massry S.G., Ritz E., Weidmann P. (1985). Prevention of intra- and postdialytic hypertensive crises by captopril.. Contributions to Nephrology..

[40] Zanoli L., Lentini P., Briet M. (2019). Arterial stiffness in the heart disease of CKD.. J. Am. Soc. Nephrol..

[41] Kaur J., Young B., Fadel P. (2017). Sympathetic overactivity in chronic kidney disease: Consequences and mechanisms.. Int. J. Mol. Sci..

[42] Neumann J., Ligtenberg G., Klein I.I., Koomans H.A., Blankestijn P.J. (2004). Sympathetic hyperactivity in chronic kidney disease: Pathogenesis, clinical relevance, and treatment.. Kidney Int..

[43] Vink E.E., de Jager R.L., Blankestijn P.J. (2013). Sympathetic hyperactivity in chronic kidney disease: Pathophysiology and (new) treatment options.. Curr. Hypertens. Rep..

[44] Kiuchi M.G., Ho J.K., Nolde J.M. (2020). Sympathetic activation in hypertensive chronic kidney disease – A stimulus for cardiac arrhythmias and sudden cardiac death?. Front. Physiol..

[45] Wever R., Boer P., Hijmering M. (1999). Nitric oxide production is reduced in patients with chronic renal failure.. Arterioscler. Thromb. Vasc. Biol..

[46] Abraham P.A., Macres M.G. (1991). Blood pressure in hemodialysis patients during amelioration of anemia with erythropoietin.. J. Am. Soc. Nephrol..

[47] Cocchi R., Degli Esposti E., Fabbri A. (1999). Prevalence of hypertension in patients on peritoneal dialysis: Results of an Italian multicentre study.. Nephrol. Dial. Transplant..

[48] Gokal R. (1999). Fluid management and cardiovascular outcome in peritoneal dialysis patients.. Semin. Dial..

[49] Günal A.I., Ilkay E., Kirciman E., Karaca I., Dogukan A., Celiker H. (2003). Blood pressure control and left ventricular hypertrophy in long-term CAPD and hemodialysis patients: A cross-sectional study.. Perit. Dial. Int..

[50] Riella M.C. (2002). Blood pressure control in dialysis patients: HD vs CAPD.. Nefrología.

[51] Krediet R.T. (2018). Ultrafiltration failure is a reflection of peritoneal alterations in patients treated with peritoneal dialysis.. Front. Physiol..

[52] Teitelbaum I. (2015). Ultrafiltration failure in peritoneal dialysis: A pathophysiologic approach.. Blood Purif..

[53] Mehrotra R., Devuyst O., Davies S.J., Johnson D.W. (2016). The current state of peritoneal dialysis.. J. Am. Soc. Nephrol..

[54] Lingens N., Soergel M., Loirat C., Busch C., Lemmer B., Schärer K. (1995). Ambulatory blood pressure monitoring in paediatric patients treated by regular haemodialysis and peritoneal dialysis.. Pediatr. Nephrol..

[55] Coomer R.W., Schulman G., Breyer J.A., Shyr Y. (1997). Ambulatory blood pressure monitoring in dialysis patients and estimation of mean interdialytic blood pressure.. Am. J. Kidney Dis..

[56] Velasquez M.T., Lew S.Q., von Albertini B., Mishkin G.J., Bosch J.P. (1997). Control of hypertension is better during hemodialysis than during continuous ambulatory peritoneal dialysis in ESRD patients.. Clin. Nephrol..

[57] Tonbul Z., Altintepe L., Sözlü Ç., Yeksan M., Yildiz A., Türk S. (2002). Ambulatory blood pressure monitoring in haemodialysis and continuous ambulatory peritoneal dialysis (CAPD) patients.. J. Hum. Hypertens..

[58] Yao Y.H., Fu C.H., Ho S.J. (2012). Peritoneal dialysis as compared with hemodialysis is associated with higher overhydration but non-inferior blood pressure control and heart function.. Blood Purif..

[59] Zhao Y. (2023). Comparison of the effect of hemodialysis and peritoneal dialysis in the treatment of end-stage renal disease.. Pak. J. Med. Sci..

[60] Vaios V., Georgianos P.I., Liakopoulos V., Agarwal R. (2019). Assessment and management of hypertension among patients on peritoneal dialysis.. Clin. J. Am. Soc. Nephrol..

[61] Devuyst O. (2021). Assessing transport across the peritoneal membrane: Precision medicine in dialysis.. Perit. Dial. Int..

[62] Zhou D., Lei H., Wu S. (2023). Influencing factors for residual kidney function in incident peritoneal dialysis patients: A systematic review and meta-analysis.. Ren. Fail..

[63] Wang M., Obi Y., Streja E. (2018). Impact of residual kidney function on hemodialysis adequacy and patient survival.. Nephrol. Dial. Transplant..

